# Identification and validation of *PROM1* and *CRTC2* mutations in lung cancer patients

**DOI:** 10.1186/1476-4598-13-19

**Published:** 2014-01-31

**Authors:** Yanqi He, Yalun Li, Zhixin Qiu, Bin Zhou, Shaoqin Shi, Kui Zhang, Yangkun Luo, Qian Huang, Weimin Li

**Affiliations:** 1 Department of Respiratory Medicine, West China Hospital of Sichuan University, 37 Guoxue Xiang, Chengdu, Sichuan 610041, China; 2 Laboratory of Molecular Translational Medicine, West China Institute of Women and Children’s Health, The Key Laboratory of Obstetric & Gynecologic and Pediatric Diseases and Birth Defects of Ministry of Education, West China Second University Hospital, Sichuan University, Chengdu 610041, China; 3 Department of Immunology, West China School of Preclinical and Forensic Medicine, Sichuan University, Chengdu 610041, China; 4 Department of Forensic Biology, West China School of Preclinical and Forensic Medicine, Sichuan University, Chengdu 610041, China; 5 Department of Radiation Oncology, The Second People’s Hospital of Sichuan, Chengdu 610041, China; 6 Department of Clinic Laboratory, Chengdu Tumor Hospital, Chengdu 610041, China

**Keywords:** Lung cancer, Gene mutation, *PROM1*, *CRTC2*, Whole genome exome sequencing

## Abstract

**Background:**

Genetic alterations could be responsible lung cancer, the leading cause of worldwide cancer death.

**Methods:**

This study investigated gene mutations in a Han Chinese family of lung cancer using the whole genome exome sequencing and subsequent Sanger sequencing validation and then confirmed alteration of prominin 1(*PROM1*) and cyclic AMP-response element binding protein-regulated transcription co-activator2 (*CRTC2*) in blood samples of 343 sporadic lung cancer patients vs. 280 healthy controls as well as in 200 pairs of lung cancer and the corresponding normal tissues using PCR-restriction fragment length polymorphism and directed DNA sequencing of PCR products.

**Results:**

The data showed *PROM1* (p. S281R) and *CRTC2* (p. R379C) mutations, in 5 and 2 cases of these 343 sporadic lung cancer patients, respectively. Notably, these mutations were absent in the healthy controls. Furthermore, in the 200 lung cancer and the matched normal tissues, *PROM1* mutation occurred in 3 patients (i.e., one squamous cell carcinoma and two adenocarcinomas) with a mutation frequency of 1.5%, while *CRTC2* mutation occurred in 5 patients (two squamous cell carcinomas and three adenocarcinomas) with a mutation frequency of 2.5%.

**Conclusions:**

The data from the current study demonstrated novel *PROM1* and *CRTC2* mutations*,* which could promote lung cancer development.

## Background

Lung cancer is a significant worldwide health problem, accounting for 13% (1.6 million) of the total cancer cases and 18% (1.4 million) cancer deaths [1]. In China, data from the most recently retrospective sampling survey showed that the rate of lung cancer death rate was 30.83/10^5^ (41.34/10^5^ for men and 19.84/10^5^ for women) [2]. The overall five year survival rate of lung cancer remains approximately 15% worldwide, driven by the majority of cases diagnosed at advanced stages, resulting in non-resectable lesions [1]. Tobacco smoking is the predominant risk factor for both small cell lung cancer and non-small cell lung cancer (NSCLC) [3]. However, to date, the defined pathogenesis of lung cancer remains to be determined because only a small fraction of tobacco smokers have developed lung cancer, but a certain percentage of lung cancer patients are never smokers, indicating that there is individual variation in cancer susceptibility in the general population and there are other factors, such as genetic factors or host contribution to a predisposition of lung cancer development [4]. Another risk factor in lung cancer development is familial aggregation and history, which associated with 73% increase (95% confidence interval [95%CI]: 50%-100%) in lung cancer risk in women and 71% increase (95%CI: 49-96%) in men [5]. Individuals with the first-degree relative lung cancer had a 1.51-fold increase in lung cancer risk compared to individuals without a family history (95%CI: 1.39-1.63) [6]. Thus, lung cancer risk models using epidemiologic data have been developed and the most parsimonious models for both ever and never smokers include a family history as a lung cancer risk [7].

Indeed, lung cancer can be aggregated in the family and inherited and appears to be the result of an interaction of multiple genes and environmental factors. Thus, search for a gene or genes associated with susceptibility is urgently needed. Previous studies showed that several tumor suppressor genes were inactivated in lung cancer patients, including *TP53*[8]*RB1*[9,10], and *PTEN*[11], while infrequent activating mutations or amplifications of *PIK3CA*, E*G*FR and *KRAS*, and *MYC* did occur in certain lung cancer patients [12]. Recent genome-wide association studies (GWAS) identified some loci that associated with lung cancer development [13,14]. Chromosomal region 15q24-25.1, containing nicotinic acetylcholine receptor sub-unit genes, has been associated with increased risk of lung cancer in ever smokers [13,14]. Linkage analysis in families with aggregation of lung cancer showed a region on chromosome 6q23-25 associated with risk of lung cancer [15]. These data plus data among individuals with a family history of lung cancer indicate that the relative risk of lung cancer associated with markers in this region is much higher in familial cases compared to the relative risk observed among sporadic cases [16,17].

Thus, in this study, we detected gene mutations in a Han Chinese family of lung cancer using the whole genome exome sequencing [18] and subsequent Sanger sequencing validation and then confirmed these gene alterations in blood samples of 343sporadic lung cancer patients vs. 280 healthy controls as well as in 200 pairs of lung cancer and the corresponding normal tissues using PCR-restriction fragment length polymorphism and directed DNA sequencing of PCR products.

## Results

### Detection of PROM1 T/G and CRTC2 G/A mutations in members of lung cancer family using whole genome Exome sequencing

In this study, we performed whole genome Exome sequencing on genomic DNA samples of the four affected and unaffected relatives in this lung cancer family (Table 1). To identify potential genetic variants associated with lung cancer development, we generated an average of 4.9 Gb of DNA sequence with 50X average coverage per individual as single-end, 90-bp reads and the fraction of effective bases on target was about 50% with a minimum 54-fold of average sequencing depth on the target. At this depth of coverage, more than 97% of the targeted bases were sufficiently covered to pass the thresholds of variant calling. We then compared the variants with the Han Chinese Beijing SNPs from dbSNP132 and the 1000 Genome Project. This generated a total of 57 genetic variants (including 55 non-synonymous SNPs and 2 splice) that were shared by the two patients, but absent in the two healthy members, which were predicted to potentially have a functional impact on the gene expression. After that, we directly sequenced PCR-products to validate these 57 variants and obtained accuracy of 67% (38/57) for those variants. We found that mutations of *PROM1* and *CRTC2* in two lung cancer patients of family cases, but absent in the two healthy members (Table 1).

**Table 1:** **Clinicopathological features of the lung cancer family with *PROM1 *** **and *CRTC2 *** **mutations**

**Patient ID**	**Gender**	**Age (years)**	**Alcohol consumption**	**Tobacco smoking**	**Pack years**	**Medical history**	**Histological type**	**TNM (Stage)**	**Tumor differentiation**	***PROM1 *** **mutation**		***CRTC2 *** **mutation**	
										Nucleotide change	Amino acid change	Nucleotide change	Amino acid change
II4	Male	65	Frequent	Former	1095	COPD, bronchiectasis	Adenocarcinoma	T1aN0M0	Moderate	841 T > G	S281R	1135G > A	R379C
III1	Male	51	Frequent	Former	365	None	Adenocarcinoma	T1bN0M0	Poor-moderate	841 T > G	S281R	1135G > A	R379C
III4	Female	37	Never	Never		None	N/A	N/A	N/A	None	None	None	None
IV1	Male	10	Never	Never		None	N/A	N/A	N/A	None	None	None	None

### Validation of genetic variants

**Figure 1:**
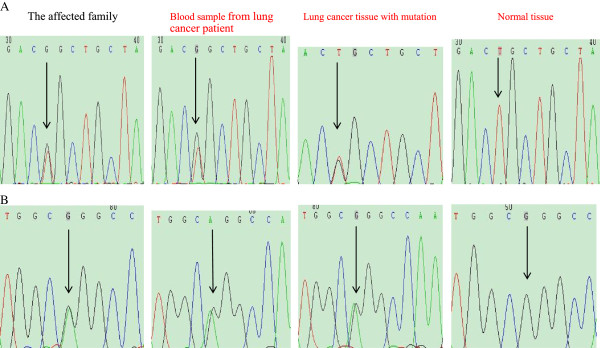
**Somatic gene mutations in III-1 patient. A**, *PROM1*mutation. **B**, *CRTC2* mutation. Tumor genomic DNA of the affected family, genomic DNA of blood sample from lung cancer patient, genomic DNA from lung cancer and the matched normal tissues.

To confirm the 38 validated genetic variants, we performed PCR-restriction fragment length polymorphism (PCR-RFLP) analysis of blood samples from 343 unrelated sporadic lung cancer patients and 280 healthy controls (Table 2). This cohort of 343 lung cancer patients had a mean age of 62.04 years old (ranged between 30 and 84 years) with 63.56% of men and 36.44% women, whereas the 280 control individuals had a mean age of 59.98years old (ranged between 35 and 80 years) with 69.64% men and 30.64% women. Age and gender between cases and controls were well balanced. We found mutations in *PROM1* and *CRTC2* in these two family NSCLC cases (Figure 1 and Table 1). *PROM1* mutation was a T to G change, resulting in an S281R amino acid change. The *PROM1* mutation was validated in 5 samples, including 3 adenocarcinomas (ADC) and 2 squamous carcinomas (SCC), with mutation rate of 1.4% (Table 3). Moreover, *CRTC2* mutation was validated with a G to A change, resulting in an R379C amino acid change (Table 3) in two lung cancer samples, including two ADCs, with mutation rate of 0.5%. However, both gene mutations were absent in these 280 controls.

**Table 2:** Clinicopathological features of 343 sporadic NSCLC cases and 280 healthy controls

	**Lung cancer**	**Control**
Characteristics	N(%)	N(%)
Age in years: median	62.04 ± 10.86	59.98 ± 9.65
Gender		
Female	125(36.44)	85(30.36)
Male	218(63.56)	195(69.64)
Smoking status		
Never	68(19.82)	N/A
Ever	107(31.20)	N/A
N/A	168(48.98)	N/A
Pack years (median, range)	432(20–732)	N/A
Histological types		
Adenocarcinoma	120(34.98)	
Squamous cell carcinoma	204(59.48)	
Adenosquamous carcinoma	8(2.33%)	
Others	11(3.21)^a^	
Clinical stage^b^		
I	76(22.89)	
II	58(17.47)	
III	91(27.41)	
IV	107(32.23)	
Tumor differentiation^b^		
Poor	204(61.44)	
Moderate and poor-moderate	121(36.45)	
Well and moderate-well	7(2.11)	
Lymph node metastasis		
Negative	154(44.90)	
Positive	189(55.10)	

^a^ including: 11 small cell lung cancer; ^b^for non-small cell lung cancer.

**Table 3:** **Clinicopathological features of patients with *PROM1 *** **and *CRTC2 *** **mutations**

**Patient**	**Gender**	**Age (yrs.)**	**Type**	**T**	**N**	**M**	**Stage**	**Differentiation**	**Tumor size (cm)**	**Family history**	**Smoking status**	**Pack years**
*PROM1*												
LC^a^-15	M	58	ADC	1a	0	0	IA	Poor	1.2 × 1 × 0.8	No	Never	
LC-116	M	70	SCC	4	0	0	IIIA	Poor-moderate	13.5 × 12 × 6	Yes	Current	182
LC-122	F	59	ADC	2a	1	1b	IV	Poor	4.3 × 2.3 × 3	No	Never	
LC-215	M	68	SCC	2b	1	0	IIB	Poor	6.1 × 4.9 × 5	No	Current	546
LC-275	F	54	ADC	4	2	1a	IV	Poor	17 × 11 × 4.1	No	Never	
LCT^b^-17	M	58	ADC	2a	1	0	IIA	Moderate	4 × 3.5 × 3.5	No	Current	182
LCT-51	M	61	SCC	1a	0	0	IA	Poor	2 × 1.5 × 1.4	No	Current	364
LCT-181	F	56	ADC	2a	1	0	IIA	Moderate-well	4 × 3 × 3	No	Never	
*CRTC2*												
LC-207	M	72	ADC	2a	0	0	IB	Moderate	2.3 × 3.4 × 2	No	Current	365
LC-250	F	65	ADC	2a	1	0	IIA	Poor-moderate	4 × 4 × 3.5	No	Never	
LCT-81	F	57	ADC	2a	0	0	IB	Poor-moderate	3 × 2.5 × 2.5	No	Never	
LCT-94	M	69	SCC	1b	0	0	IA	Poor	2.5 × 1.5 × 1.0	No	Current	500
LCT-111	M	52	SCC	2a	1	0	IIA	Poor-moderate	4 × 3.5 × 2.5	No	Current	280
LCT-160	M	41	ADC	2a	1	0	IIA	Moderate	4 × 3.5 × 4	Yes	Never	
LCT-171	F	69	ADC	2a	0	0	IB	Poor-moderate	4 × 4 × 3	No	Never	

^a^blood sample of lung cancer; ^b^tissue sample of lung cancer and matched normal lung tissue.

ADC, adenocarcinoma; SCC, squamous cell carcinoma.

In addition, we further verified *PROM1* and *CRTC2* mutations in an additional 200 pairs of lung cancer and the matched normal tissue specimens (Table 4). We found *PROM1* mutation in 3 pairs of lung cancers and normal tissues, including 1 SCC and 2 ADCs with mutation frequency being 1.5% and *CRTC2* mutation in 5 pairs of lung cancers and normal tissues, including 3 ADCs and 2 SCCs with mutation frequency being 2.5%. All mutations were further confirmed by direct DNA sequencing of PCR products (Figure 1 and Table 3).

**Table 4:** Clinicopathological features of these 200 sporadic lung cancer tissues

	**Lung cancer**
Characteristics	N(%)
Age in years: median	57.93 ± 10.480
Gender	
Female	56(28.00)
Male	144(72.00)
Smoking status	
Never	92(46.00)
Ever	108(54.00)
Pack years (median, range)	741.94(10–3000)
Histological type	
adenocarcinoma	95(47.50)
squamous cell carcinoma	93(46.50)
adenosquamous carcinoma	10(5.00)
others	2(1.00)^a^
Clinical stage^b^	
I	54(27.27)
II	56(28.28)
III	70(35.35)
IV	18(9.10)
Tumor differentiation^b^	
Poor	92(46.47)
Moderate and poor-moderate	100(50.51)
Well and moderate-well	6(3.02)
Lymph node metastasis	
Negative	98(49.00)
Positive	102(51.00)

^a^including: 2 neuroendocrine carcinoma.

^b^for non-small cell lung cancer.

## Discussion

In the current study, we sequenced and compared the whole genome coding regions of genes in two lung cancer patients and two healthy family members and then filtered the benign changes using public databases, including the 1000 Genome Project and dbSNP132. Use of the second-generation sequencing technique produced a high level of coverage with higher accuracy and allows more regions of a genome to be sequenced in very cost effective manner. We successfully identified *PROM1* and *CRTC2* mutations. Our data are novel and mutation of these two genes had not been previously reported in lung cancer. After that, we confirmed our data in blood samples of 343 lung cancer patients, but not in 280 healthy controls. These two gene mutations were not reported in the 1000 Genomes data and dbSNP132.

*PROM1* is localized at chromosome 4p15.32 and contains 27 exons to code CD 133 protein. To date, the precise functions of CD133 protein are unknown although it is a member of five-transmembrane glycoproteins. The latter proteins specifically localize to plasma membrane protrusions. CD133 protein has two short N (extracellular)- and C (cytoplasmic)-terminal tails, and two large N-glycosylated extracellular loops (between TM2 and -3,and TM4 and -5). *PROM1* can translate into 7 isoforms of CD133 protein through the alternative splicing in human tissues and the alternatively spliced exons in the coding region only affect the short N- and the C-terminal domains [19,20]. CD133 is expressed in various normal and stem cells; thus, it was classified as a marker of primitive hematopoietic and neural stem cells. Recently, rapidly accumulated evidence indicates that CD133 had been described as the most important marker inherent to a number of types of cancer stem cells (CSCs) [21-24]. These CSCs can initiate the tumors with performing unique functions, such as asymmetric division, self-renewal, drug resistance and quiescence [25]. A previous study reported that a rare population of tumorigenic cells in small cell and non-small cell lung cancer expressed CD133 protein [26]. Lung cancer contained a rare population of CD133^+^ CSCs able to self-renew and generated an unlimited progeny of non-tumorigenic cells [27]. Cui *et al.* investigated several human lung cancer cell lines and found that CD133 was a marker for the small cell lung cancer and had stem cell-like features, such as self-renewal, differentiation, proliferation and tumorigenic capacity [25]. *PROM1* mutations, including p.R373C, p.Q576X, p.G614fsX626, and p.Y452fsX12, were associated with a heterogeneous group of inherited retinal disorders with autosomal recessive and dominant inheritance patterns [26,28]. In our current study, we identified somatic *PROM1* mutation (p.S281R) in 8/550 lung cancer patients and this novel *PROM1* mutation was a T to G change, resulting in an S281R amino acid change. All of the 8 patients had non-small cell lung cancer (i.e., 5 adenocarcinomas and 3 SCCs), five of which developed lung cancer at the age younger than 60 years old and two of which were classified as stage IV disease with poor differentiation. The mutation of *PROM1* is novel in lung cancer and further study will investigate the role of CD133 protein in lung cancer development and progression.

Furthermore, *CRTC2* is localized at chromosome 1q21.3 and codes a 693 amino acid protein, namely CRTC2 [29]. CRTC2 protein contains an N-terminal GREB-binding domain (CBD), a central regulatory (REG) domain, a splicing domain (SD), and a C-terminal domain (TAD). CRTCs have shown to induce expression of cyclic AMP-responsive genes [30]. CRTCs are sequestered in the cytoplasm through phosphorylation-dependent interactions with 14-3-3 proteins. In the absence of AMPK activity, CRTC2 is dephosphorylated and translocated into the nucleus where it associated with CREB and induces expression of the target genes [31]. It has reported that CRTC2 was able to regulate gluconeogenesis by integrating calcium and metabolic signaling through calcineurin and AMPK/SIK kinase families [32]. Another study identified LKB1 as an essential activator for the AMPK gene family and a key regulator for CRTC2 transcriptional activity [33], while Shaw *et al.* demonstrated that SIKI may participate in mediating LKB1 tumor suppressor activity [34]. Ji *et al.* found that LKB1 mutations are detected in human tumor samples, including lung cancer [35]. Komiya *et al.* found enhanced activity of CRTC1 in LKB1-null lung cancer [36]. Brown *et al.* reported CRTC2 play an important role in breast cancer of postmenopausal women [37]. In our current study, we found somatic *CRTC2* mutation in 7/550 lung cancer patients. This novel mutation site could change G to A and result in an R379C amino acid change. All of these 7 patients had stage I and II non-small cell lung cancer (i.e., 5 adenocarcinomas and 2 SCCs) without metastasis. However, it remains unknown what function of CRTC2 protein plays in lung cancer and further studies are warranted.

## Conclusions

In summary, our current study identified and confirmed novel mutations of *PROM1* and *CRTC2*inNSCLC patients. Since the numbers affected patients are too small, we don’t know whether these mutations can affect survival of patients. Further studies will investigate the role of these two proteins in lung cancer development and progression.

## Methods

### Study population

**Figure 2:**
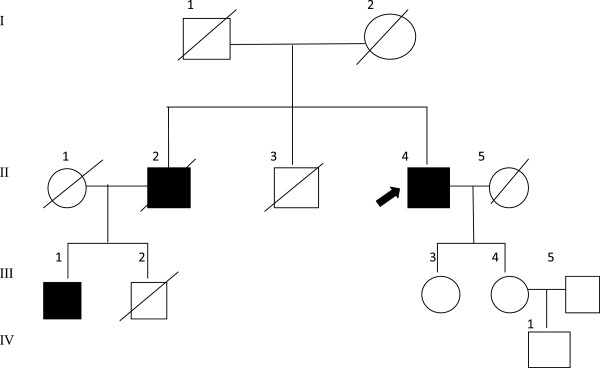
**Pedigree of this lung cancer family.** Hepatocarcinoma occurred in I-2, II-3, and III-2; lung cancer in II-2, II-4, and III-1; and gastric cancer in II-5.

This study included a four-generation Chinese Han family with lung cancer cases that had 13(6 living) family members (Figure 2; Table 1). The proband (II-4) was a 65-year-old man who had been diagnosed with adenocarcinoma of the lung in 2012 with a 10-year history of chronic obstructive pulmonary disease (COPD) and bronchiectasis. He had smoked for the past 38 years with approximately 1095 pack per year. Biochemical examination showed an increased CEA (8.97 ng/mL, normal < 5 ng/mL), while CT scanning showed a single nodule with 1.5 × 1.5 × 1.0 cm in the apicoposterior segment of the left upper lobe. This proband’s 51-year-old nephew (III-1) presented with cough, hemoptysis and dyspnea. CT scanning confirmed unilateral mass in the superior segment of the right lower lobe. In 2009, he underwent an open lung lobectomy and the result of histopathological examination confirmed as lung adenocarcinoma. In this family, other members also died of lung cancer (II-2), hepatocarcinoma (I-2, II-3, III-2), or gastric cancer (II-5).

Furthermore, to confirm gene mutations in lung cancer of this family members, we recruited 343sporadic lung cancer patients vs. 280 healthy controls as well as 200 pairs of lung cancer and the corresponding normal tissues from The West China Hospital of Sichuan University, The Second People’s Hospital of Sichuan, and The Seventh People’s Hospital of Chengdu, Chengdu, China between March 2010 and June 2012. All of the sporadic lung cancer patients and the healthy controls were non-related and were of Chinese Han ethnicity (Table 2). Those with secondary lung cancer or other serious disease were intentionally excluded. The diagnosis was confirmed by histopathological examination of the resected or biopsy tissue specimens in all cases. The demographic and clinical information were collected, including sex, age at admission, tobacco smoking, histological diagnosis, tumor-node-metastasis clinical stage according to the American joint committee on cancer 2010 guidelines [38,39]. The study was approved by the Ethics Committee of West China Hospital of Sichuan University and all participants provided informed consent.

### DNA samples

Peripheral blood collected from patients and controls into an anticoagulation tube and stored at -80°C until use. Genomic DNA was then extracted using a commercial DNA isolation kit from Bioteke (Beijing, China) according to the manufacturer’s instructions. Tumor tissues were fixed in formalin and embedded in paraffin and then sectioned for hematoxylin and eosin staining. Pathologists then identified areas of adequate tumor lesion (>70%) for DNA extraction. The normal lung tissue of the patients obtained during surgical resection represented lung tissue located more than 5 cm from tumor lesions. Genomic DNA was extracted using the Tissue Kit (Qiagen, Valencia, CA) according to the manufacturer’s protocol.

### Whole genome exome capture and massively parallel DNA sequencing

We then performed a whole genome exome sequencing to identify the disease-causing genetic variant for this lung cancer family. Briefly, 15 μg of genomic DNA samples from each of these four individuals (II-4, III-1, III-4, and IV-1) were separately sheared into approximately 200 to 300 bp DNA fragments by Covaris, and adapters were then ligated to both ends of the resulting DNA fragments and amplified by ligation-mediated PCR (LM-PCR), purified, and hybridized to the NimleGen 2.1 M human exome array for enrichment, non-hybridized fragments were then washed out. Both non-capture and capture LM-PCR products were subjected to quantitative PCR to estimate the magnitude of enrichment. Each capture library was then loaded on Hiseq2000 platform, and high-throughput sequencing was performed for each captured library independently to ensure that each sample at least 50-fold coverage. Raw image files were processed by Illumina base calling Software 1.7 for base calling with default parameters and the sequences of each individual were generated as 90 bp paired-end reads.

Sequencing reads were aligned to the human genome (hg19) as the reference genome sequence, together with its gene annotation that was downloaded from the UCSC database (http://genome.ucsc.edu/). Single nucleotide polymorphisms (SNPs) were identified by *SOAPsnp*[40] and small insertion/deletions (InDels) were detected by *SAM tools*[41]. SNPs and indels were defined based on satisfaction of the following: i) The consensus quality score was bigger than Q20 (the quality score is a Phred score, generated by the program SOAPsnp12, quality score 20 represents 99% accuracy of a base call); ii) The depth of supported reads at that locus was no less than 4X and no more than 1000X; and iii)The distance between two SNPs no less than 5. The changes that were shared in the two affected individuals, but absent in the two unaffected individuals were obtained by further comparison of the variants of each of the four individuals. All changes were filtered against the Han Chinese Beijing SNPs in the dbSNP132, and the 1000 Genome Project (February 28, 2011 releases for SNPs and February 16, 2011 releases for indel http://www.1000genome.org). All variants were then confirmed by direct DNA sequencing of polymerase chain reaction (PCR) product (BigDye® Terminator v3.1 Cycle Sequencing Kits; Applied Biosystems, Foster City, CA).

### Validation of gene mutations

To further verify the genetic variants identified in these two lung cancer patients sporadic lung cancer patients and the healthy controls, we performed PCR according to the manufacturer’s instructions. Primers were synthesized by using the on-line software (http://frodo.wi.mit.edu/primer3/) for PCR amplification of variants identified via exome sequencing. The primers of *PROM1* mutation were 5′-GACCGCAGGCTAGTTTTCAC-3′ and 5′-CTTGCAGTGTGTCCCTCTCA-3′ and the primers of *CRTC2* mutation were5′-GAGGAGGAAGAGGAGGAGGA-3′ and 5′-CTAAGCAATCCCAACCTCCA-3′.PCR products were then digested overnight with specific restriction enzyme MspI and separated by a 6% polyacrylamide gel electrophoresis and stained with 1.5 g/l of argent nitrate to visualize both *PROM1* T/G and *CRTC2* G/A mutations. The genotypes and all mutations were confirmed by the DNA sequencing analysis (BigDye® Terminator v3.1 Cycle Sequencing Kits; Applied Biosystems, Foster City, CA). Approximately 10% of the cases were randomly selected for the repeated assays and the results were 100% concordant.

## Competing interests

The authors declare that they have no competing interests.

## Authors’ contributions

YH and YL carried out the molecular genetic studies, participated in the sequence alignment and drafted the manuscript. ZQ and SS carried out the immunoassays. KZ, YL and QH participated in the sequence alignment. YH and BZ participated in the design of the study and performed the statistical analysis. WL conceived of the study, and participated in its design and coordination and helped to draft the manuscript. All authors read and approved the final manuscript.
